# Mobile Phone Apps for University Students With Hazardous Alcohol Use: Study Protocol for Two Consecutive Randomized Controlled Trials

**DOI:** 10.2196/resprot.4894

**Published:** 2015-12-22

**Authors:** Anne H Berman, Mikael Gajecki, Morgan Fredriksson, Kristina Sinadinovic, Claes Andersson

**Affiliations:** ^1^Center for Psychiatry ResearchDepartment of Clinical NeuroscienceKarolinska InstitutetStockholmSweden; ^2^Liquid Media ABStockholmSweden; ^3^Department of CriminologyMalmö UniversityMalmöSweden

**Keywords:** randomized controlled trial, universities, alcohol abuse, prevention, mobile phone, eHealth, mHealth

## Abstract

**Background:**

About 50% of university students overconsume alcohol, and drinking habits in later adulthood are to some extent established during higher educational studies. Several studies have demonstrated that Internet-based interventions have positive effects on drinking habits among university students. Our recent study evaluated two mobile phone apps targeting drinking choices at party occasions via personalized feedback on estimated blood alcohol concentration (eBAC) for students with hazardous drinking. No changes in drinking parameters were found over a seven-week period apart from an increase in number of drinking occasions among men for one of the apps tested. Up to 30% of the study participants drank at potentially harmful levels: higher than the national recommended number of standard drinks per week (a maximum of 9 for women and 14 for men) in Sweden.

**Objective:**

(1) To evaluate improved versions of the two mobile phone apps tested in our prior trial, in a new, 3-armed randomized controlled trial among university students with at least hazardous drinking habits according to the Alcohol Use Disorders Identifications Test (AUDIT; Study 1). (2) After 6 weeks, to target study participants showing alcohol consumption higher than the national recommended levels for standard drinks per week by offering them participation in a second, 2-armed randomized trial evaluating an additional mobile phone app with skill enhancement tasks (Study 2). (3) To follow participants at 6, 12 and 18 weeks after recruitment to Study 1 and at 6 and 12 weeks after recruitment to Study 2.

**Methods:**

Two randomized controlled trials are conducted. Study 1: Students are recruited at four Swedish universities, via direct e-mail and advertisements on Facebook and student union web sites. Those who provide informed consent, have a mobile phone, and show at least hazardous alcohol consumption according to the AUDIT (≥6 for women; ≥8 points for men) are randomized into three groups. Group 1 has access to the Swedish government alcohol monopoly’s app, Promillekoll, offering real-time estimated eBAC calculation; Group 2 has access to a Web-based app, PartyPlanner, developed by the research group, offering real-time eBAC calculation with planning and follow-up functions; and Group 3 participants are controls. Follow-up is conducted at 6, 12 and 18 weeks. Study 2. Participants who at the first 6-week follow-up show drinking levels higher than 9 (W) or 14 (M) standard drinks (12 g alcohol) per week, are offered participation in Study 2. Those who consent are randomized to either access to a skills training app, TeleCoach or to a wait-list control group.

**Results:**

Latent Markov models for Study 1 and mixed models analyses for Study 2 will be performed. Study 2 data will be analyzed for publication during the spring of 2016; Study 1 data will be analyzed for publication during the fall of 2016.

**Conclusions:**

If mobile phone interventions for reducing hazardous alcohol use are found to be effective, the prospects for positively influencing substance use-related health among university students can considerably improve.

**Trial Registration:**

ClinicalTrials.gov http://clinicaltrials.gov/ct2/show/NCT02064998 (Archived by WebCite at http://www.webcitation.org/6dy0AlVRP)

## Introduction

Research studies have shown that consumption of alcohol is higher during the years at university than at any other age [1]. Furthermore, it appears that students at college and university establish their future adult alcohol habits [2]. Individual, social and environmental factors affect this development [3]. Several studies have demonstrated that effective intervention approaches, both at individual and group levels, are available to reduce hazardous drinking among students. These methods are well documented internationally [4,5] and as well as nationally in a meta-analysis [6].

A major behavior change component for reducing problematic alcohol use in general is screening with brief intervention (SBI), where the primary goal is to moderate consumption to sensible levels and to eliminate harmful drinking practices in order to reduce negative outcomes of drinking [7]. At minimum, SBI includes only screening of drinking habits, followed by clear and direct feedback. It can also include detailed conversations covering setting up goals, use of behavioral modification techniques, self-help exercises, and continual reinforcement [8]. For university students, effective interventions are termed brief motivational interventions (BMIs), providing personalized feedback on individual drinking habits and their consequences, based on self-monitoring, as well as exploration of motives for using alcohol. Specific behavior change components include the personalized feedback aspect, particularly normative feedback, in relation to students in the same university context; studies of feedback on Blood Alcohol Concentration (BAC) have shown mixed support [9].

### New Technology

Despite availability of interventions, only a small percentage of students seek help for problem drinking [10]. College students with heavy episodic drinking (HED) at least once a month have been found to prefer computerized methods [11]. Digital interventions for alcohol problems, regardless of delivery mode, offer small but meaningful effects [12-18]. They are as effective as alternative interventions offered face-to-face by a live counselor when compared with controls but, in direct comparisons, face-to-face interventions have been shown to be more effective [17,18].

### Mobile Apps

In recent years, mobile phones have offered constant access to hand held computers. Easy to use mobile phone apps can fill a variety of functions, such as games and other entertainment. Depending on its function, the app may or may not require an Internet connection. Apps have been used for registering weight for obesity control [19] and, in a guided version, to help users with behavioral activation in their own chosen valued direction [20]. Research on mobile phone apps for reducing alcohol consumption is in its infancy. Over 3000 apps on alcohol-related topics are available, but these usually have no therapeutic purpose and even provide incorrect information, for example regarding blood alcohol content [21]. An analysis of user experiences of 87 Android apps showed, however, that these in some cases inspire users to keep their alcohol use down [22]. A very recent content analysis of 800 alcohol apps with a focus on behavior change techniques found that the majority “implicitly or explicitly promoted the use of alcohol”. Of 61 apps coded for behavior change techniques, the most frequent techniques used (over 40%) were self-monitoring, information on negative consequences of alcohol use and positive consequences of abstinence, and personalized feedback. The analysis cites no research specifically evaluating the apps reviewed [23].

**Figure 1:**
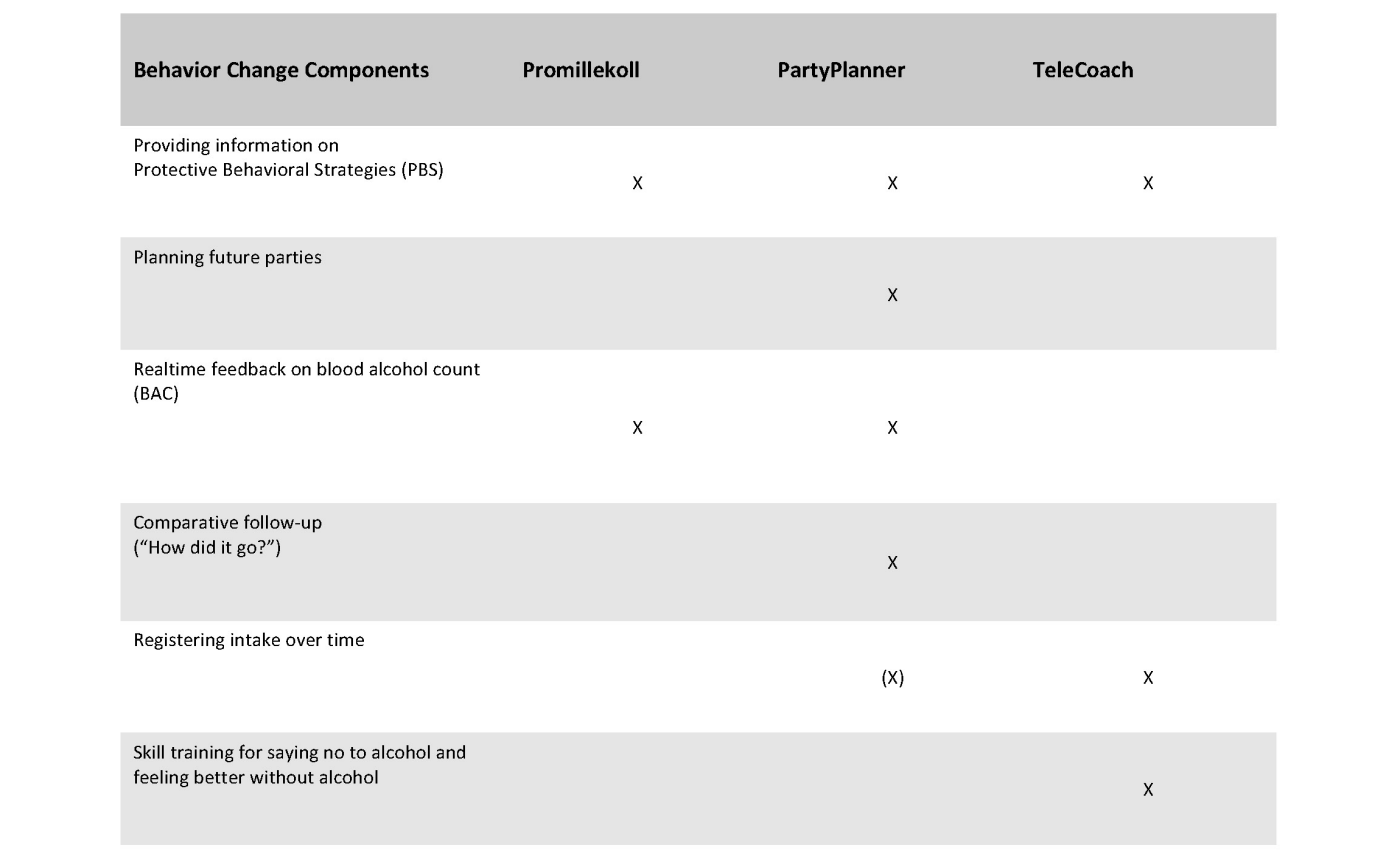
Behavior change components included in the two apps used in Study 1 and in the app used in Study 2.

Indeed, systematic research on mobile phone apps for university students has so far been very sparse [24]. One study targeted smoking and HED based on BASICS (Brief Alcohol Screening and Intervention for College Students) and found no reduction over a one-month period in HED, although students who completed more intervention modules were less likely to drink during the initial 14-day assessment period in the study [25]. In a second study, our own research group conducted a 3-armed randomized controlled trial among university students with problematic drinking, comparing the effects of 2 different apps, one offering personalized feedback on estimated blood alcohol concentration (eBAC) at live drinking occasions and the other offering planning, live monitoring of eBAC with personalized feedback, and follow-up of specific drinking occasions based on eBAC (see Figure 1 for an overview of app components). We found no changes in drinking parameters over a 7-week period apart from an *increase* in number of drinking occasions among men for one of the apps tested. Also, dropping out from the study was associated with drinking at levels higher than the recommended maximum number of standard drinks per week (9 for women and 14 for men); students who drank at higher than recommended levels comprised a sub-group of almost one-third of students assessed as having at least hazardous drinking habits according to the Alcohol Use Disorders Identification Test (AUDIT) [26].

In recent years, alcohol apps for mobile phones have seen an exponential rise in growth. Their potential is high in view of their extensive reach in the population, but the evidence for their effectiveness in reducing problematic alcohol use is lacking [27]. Questions that remain to be answered are whether it is possible to reduce hazardous or even harmful drinking with apps, as well what components an app actually needs to include in order to be effective.

### Study Aim

This research has two principal aims. First, we aim to evaluate mobile phone apps in improved, revised versions compared to our previous study [26] for university students with problematic levels of alcohol use based on AUDIT scores for at least hazardous drinking. We also aim to extend follow-up beyond 6 weeks, to 12 and 18 weeks. We hypothesize that the improved app versions may show positive effects with reduced alcohol use over time in comparison to an untreated control group, in contrast to no effects in the 6-week follow-up in our previous study [26]. Secondly, we aim to target the needs of a sub-group of university students with particularly high alcohol consumption according to national public health thresholds for unhealthy drinking, by evaluating a new mobile phone app with skills enhancement functions. We hypothesize that dropout over time will be reduced for students in this sub-group, who are offered an app addressing their specific, more extensive need of help to control drinking. Also, we hypothesize that students offered this app will reduce their drinking to a larger extent than students in a wait-list control group as well as those in an untreated control group (separately culled from the wider data set for Study 1). These aims will be addressed through two consecutive randomized trials (Trial Registration at ClinicalTrials.gov, identifier NCT02064998).

**Figure 2:**
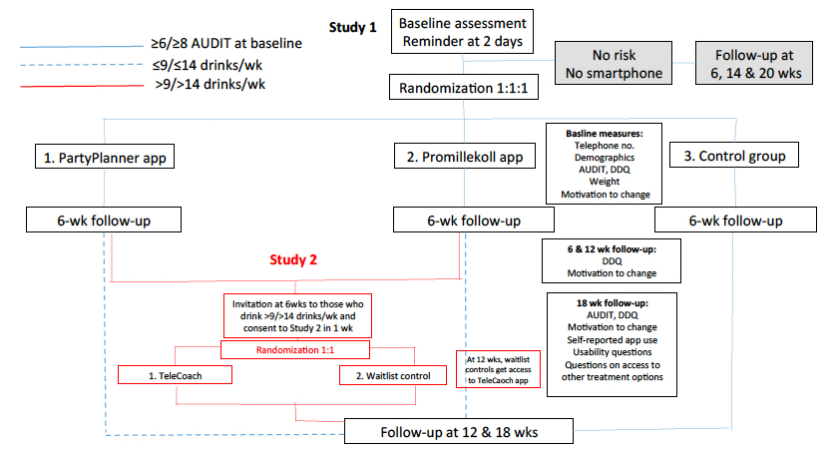
Flowchart showing study design for Studies 1 and 2, including follow-up measures.

The specific research questions are as follows:

Study 1: Among university students with at least hazardous alcohol use according to the AUDIT, does access and self-reported use of two different mobile phone apps (see Figure 2) lead to reduced alcohol consumption in comparison to an untreated, assessment only control group, at 6, 12 and 18 weeks after registration for the study?

Study 2: Among university students who drink over the recommended 9 and 14 standard drinks per week for women and men, respectively, does access and self-reported use of the TeleCoach app (see Figure 2) lead to lower proportions of individuals with consumption levels under the recommended number of drinks per week, in comparison to a wait-list control group and an untreated control group, 6 and 12 weeks after registration to Study 2 (12 and 18 weeks after registration for Study 1)?

## Design

We plan to conduct two consecutive randomized controlled studies.

As in our previous trial [26], we plan to cooperate with the student unions at Stockholm University, KTH Royal Institute of Technology and Södertörn University. Students are contacted via direct email or via advertisements on student union websites or Facebook pages. The direct emails provide information to prospective study participants on a randomized study of mobile phone mobile apps concerning alcohol habits. Prospective participants clicking on the invitation link will be asked to fill in information on age, gender, term of study, possible previous use of apps, and questions on alcohol use according to the Daily Drinking Questionnaire (DDQ) [28] and the 10-item AUDIT [29].

Any interested person completing the online registration process will be included in the study, regardless of level of alcohol use. Individuals without any hazardous drinking will thus be recruited for this research including all follow-ups, but they will not be randomized to any intervention. We will conduct a later secondary analysis of all data including students without hazardous drinking, to explore and discuss the regression to the mean phenomenon in this group [30].

Problematic alcohol use will be defined in two ways. All potential participants will have filled in the AUDIT and the DDQ. Those with AUDIT scores of ≥6 for women and ≥8 points for men will be defined as drinking alcohol at levels that are at least hazardous to their health. Those with weekly drinking levels according to the DDQ, of over 9 drinks per week for women and 14 drinks per week for men, will be defined as having elevated alcohol consumption levels. The AUDIT-based definition is the basis for recruitment to Study 1, whereas the DDQ-based definition is the basis for recruitment to Study 2.

## Procedure

A flowchart showing the procedure and interconnection between Studies 1 and 2 is shown in Figure 2.

### First Study

In Study 1, we basically repeat the design of our original study from spring 2013, in which students with problematic alcohol use were randomly allocated to one of three groups: the Swedish Alcohol Monopoly’s Promillekoll app, the research group’s PartyPlanner app or a control group. The planned study differs from the original study in two aspects. First, app content will have been updated for both apps (see below under Interventions). Secondly, the follow-up time is extended from only one follow-up at 6 weeks, to three follow-ups at 6, 12 and 18 weeks.

Participants owning a mobile phone and showing at least hazardous use of alcohol, defined as scores of ≥ 6 for women and ≥ 8 for men on the AUDIT, are randomized to a group with access to one of the two apps, or to an untreated, assessment-only control group. Participants randomized to an intervention are sent an email with a link to the respective app with instructions on how to install the app, and a brief recommendation to use the app on drinking occasions. As noted above, participants without risky alcohol use and/or without a mobile phone are excluded from randomization. All participants are informed that they will be contacted after 6, 12 and 18 weeks via email for follow-up.

### Second Study

Participation in Study 2 will be offered to students in the Study 1 intervention groups who, at 6-week follow-up, show elevated alcohol consumption in excess of 9 and 14 standard drinks a week according to the DDQ (for women or men, respectively). This level of alcohol consumption is connected to an elevated risk of harmful consequences, and is of particular concern since 1 out of 4 deaths among individuals 15-29 years old in the EU are alcohol-related [31]. These students are given feedback on their elevated consumption level with higher risk of harmful consequences. They continue to have access to the app they were randomized to in Study 1, but are randomized to one of two groups: access to an in-depth self-help TeleCoach app that the research group has developed, or to a wait-list control group who will be given access to the app at the 12-week follow-up.

All study participants who complete the final follow-up will participate in a lottery of 3 iPad devices, through a partnership with Save the Children.

### Interventions

All three app interventions used in Studies 1 and/or 2 are described below. Figure 1 shows a simplified overview of basic behavior change components included in each app.

#### Promillekoll App (Translation: “Check your BAC”)

This mobile phone app is available in iPhone and Android versions, and allows the user to register alcohol consumption in real time, giving immediate feedback on the eBAC. The app alerts the user if s/he surpasses an alcohol concentration of 0.06 percent BAC, a level where negative consequences can begin to occur, and only displays values up to 0.08 percent, in an effort to emphasize the message to users that higher eBAC levels are harmful. The app also provides separate information texts on alcohol and BAC. The app was publicly launched by the Swedish Alcohol Monopoly (Systembolaget) in the autumn of 2012 and improved a year later on the basis of the Monopoly’s own surveys and 343 student comments from our study in the spring of 2013 (unpublished data). The Promillekoll app is theoretically based on the assumption that information about one’s own real-time eBAC levels can contribute to protective cognitive and behavioral strategies. A further mechanism, congruent with the Theory of Planned Behavior (TPB) [32], is that providing information and feedback on risky levels of eBAC modifies the intention to consume alcohol. Promillekoll also offers selected specific protective behavioral strategies (PBS) [33] to maintain alcohol consumption at or below the 0.06 percent BAC level. No user data are collected.

#### PartyPlanner App

This Web-based app guides the user to plan drinking occasions so the consumption level stays below risky levels of eBAC, at about 0.06 percent. The app also allows the user to record real-time consumption and provides immediate feedback on how to adjust their consumption to maintain a healthy eBAC level to 0.06 percent or less. If the user has created a plan for a specific drinking occasion and then followed it up, the app offers a graphic visual comparison of the plan, with the logged real-time event after the actual drinking occasion. These app components are in line with the goal setting and personalized feedback components of successful BMIs [9].

**Figure 3:**
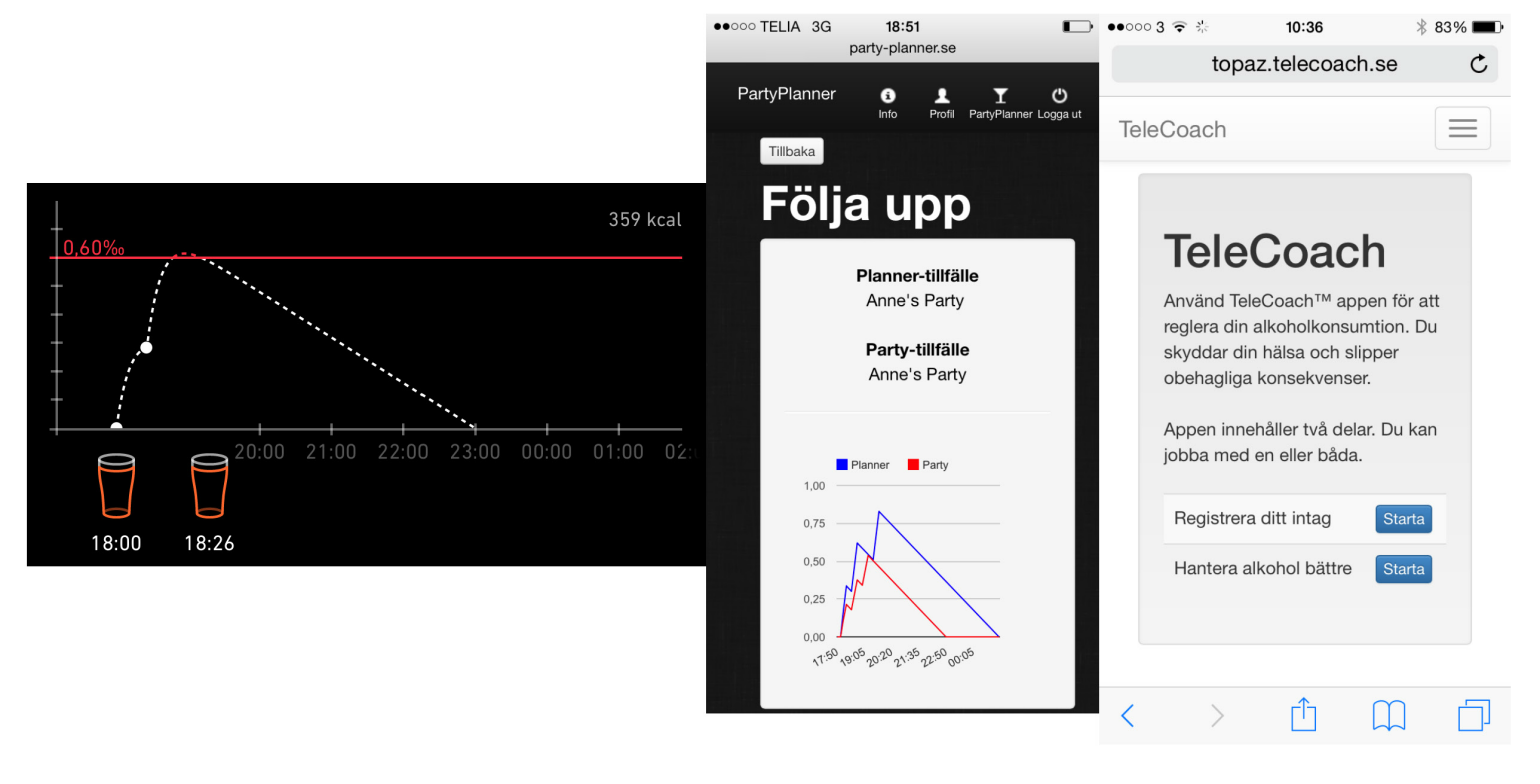
Screen dumps from each of the three apps described in the article. From left to right: Promillekoll, PartyPlanner, TeleCoach.

The app was built by the research team in the fall of 2012 and revised in the fall of 2014 for the current study. In the revised version, users receive feedback about the health benefits of lower alcohol use and potential social losses connected to over-consumption, since laboratory studies have shown that this kind of information affects men’s drinking intentions in a downward direction [34]; we focused on adapting information primarily to men because we saw a significant negative effect for men using Promillekoll in our first study, and unpublished secondary analyses suggested that men might have more difficulty using an app constructively in a social context where alcohol is consumed, than women. An example of the type of feedback given is shown in Figure 3. PartyPlanner also includes several PBS [33] to maintain alcohol consumption at a minimally harmful level.

Note: The level for non-risky eBAC is below 0.06 percent for both Promillekoll and PartyPlanner, in correlation with guidelines used in the BASICS program [35]; this level is somewhat more conservative than the 0.08 percent level correlated with the concept of binge drinking [36].

#### TeleCoach App

This app is based on an Interactive Voice Response (IVR) automated telephony intervention developed by the research group in 2009-2012 [37]. Aside from offering skill acquisition for PBS [33], it provides skill training exercises for individuals who want to reduce or end their alcohol consumption but have experienced trouble in achieving these behavioral changes. The app includes a behavioral chart, and modules on “saying no to alcohol” and “feeling better without alcohol.” The “saying no” module includes instruction on firm body language and voice, a guide to the five principles of saying no [38] and the Alcohol Abstinence Self-Efficacy Scale [39]; the “feeling better” module includes relaxation exercises, lists of positive thoughts [40] and urge surfing [38]. In this study, we will test the app version of the intervention for the first time, targeting university students who consume alcohol over the national recommendations of no more than 9 and 14 standard drinks/week for woman and men, respectively.

### Assessment

The baseline, 6, 12 and 18 week follow-ups, include the Daily Drinking Questionnaire (DDQ) [28], and all 10 questions in the AUDIT [41]. The DDQ was developed in the US and was adapted for use by students by Collins et al (1985). The instrument was translated and adapted for use in Sweden by the Department of Clinical Alcohol Research at Lund University, and has been used in previous studies of Swedish students at the university [42]. The AUDIT is an effective instrument for identifying problematic alcohol consumption. The instrument was developed by the World Health Organization (WHO) and measures consumption as well as signs of harmful use and dependency related to alcohol use. The Swedish version was translated and disseminated by researchers at Karolinska Institutet [43]. The assessment package also includes information about participants’ mobile phone number, gender and age, and possession of a mobile phone. All follow-ups also include questions about users experience interacting with the apps. The 18-week follow-up also includes questions regarding access, during the 18 weeks since registration, to other types of treatment such as additional sources of information, medication, speaking to counselors or using other apps than the one they are randomized to; more information on these questions is available in studies on minimal Web-based intervention conducted by our group [44,45]. Invitations to participate in follow-ups are sent via e-mail, where participants are asked to log on to a secure survey to complete assessments. Two email reminders are sent out during the one-week time period allowed for response.

### Power Calculation

Primary outcomes for both studies are quantity, frequency, number of binge drinking occasions as well as mean and peak eBAC, and proportion of individuals drinking over the recommended levels of 9 and 14 drinks per week, for women and men respectively.

For Study 1, in order to answer the questions on whether the use of a mobile phone app affects alcohol consumption compared with controls, 82 individuals need to be included in each of the three groups (two intervention and one control group, 246 individuals in total) in order to detect a difference in effect size 0.10 at 5% -level with a power of 80%. This is based on an assumed correlation between DDQ, pre- and post-measurement of at least 0.5. It is likely, however, that high power can be achieved since approximately 7,000 students will be invited to participate in the study. We expect at least 1200 students to agree to participate based on the 17% recruitment level achieved in our previous study [26], but we hope more will participate since we will be targeting first- and second-term university students in this research, in contrast to all registered students regardless of study level, in the previous study. About half of the recruited students are expected to have at least hazardous use and be eligible for randomization.

For Study 2, based on the same assumption as above, 82 individuals need to be included in each group (intervention and wait-list control group), 164 individuals in total. Based on our prior study [26] we expect that approximately 30% of the study participants will have an elevated risk consumption at 6-week follow-up, and estimates based on previous experiences of similar design [46] suggest that at least 50% will want to participate in the study, suggesting that adequate power will be available.

### Ethics and Time Plan

The project was approved by the regional ethical review board on March 19, 2014 (2014/278-31/2). The study commenced in early autumn 2014. Recruitment began during the last week of September, 2014. Participants who at 6-week follow-up showed harmful use of alcohol over 9 or 14 standard drinks per week for women and men, respectively, were offered participation in the second study. All participants were followed up at 6, 12 and 18 weeks after baseline recruitment to the project. Data collection was completed in the late spring of 2015. The results will be analyzed during the autumn of 2015, see below for the analysis plan.

## Results

Studies 1 and 2 will be analyzed separately. For both studies, descriptive statistics will be used to describe baseline characteristics. Analysis of variance (ANOVA) will be used to identify any baseline differences in age, AUDIT, quantity, frequency, number of binge drinking occasions, as well as mean and peak eBAC between the groups. Pearson’s chi-square tests will be used to determine differences between the groups in the gender distribution and the proportion of participants drinking more than the weekly recommendation.

For Study 1, all participants in both studies will be included in the analysis. Latent Markov models [47] will be used to maximize differing results for Study 1 participants not offered participation in Study 2, Study 1 participants who declined participation in Study 2, and Study 2 participants.

For Study 2, a linear mixed model analysis will be used to identify changes over time in alcohol consumption outcomes: quantity, frequency, and number of binge drinking occasions, mean eBAC and peak eBAC. These analyses will be conducted per protocol—that is, including only those participants who report using the app they were assigned to, and controlling for self-reported access to other treatment sources for problematic alcohol use during the study, and having accessed the publicly available Promillekoll app prior to the study. For comparison, intention to treat analyses will be performed with all participants randomized to experimental groups and retaining baseline values for as many participants as possible.

Descriptive statistics, ANOVA and Pearson’s chi-square analyses will be performed using IBM SPSS Statistics for MacOS X, Version 22 (IBM Corp). Linear mixed model analyses will be performed using Stata 13 (StataCorp). Values for averages and standard deviations are presented to three decimal places for variables where this is necessary in order to make differences visually discernible.

## Discussion

### Summary

Harmful alcohol use is a significant problem in university students. Even though effective face-to-face interventions are available, most students prefer the digital interventions developed in recent years [48]. Computer use has also undergone rapid changes, and today most interaction with computers is through mobile phones [49]. Literally thousands of alcohol apps have developed for mobile phones, but the evidence for their effectiveness in reducing problematic alcohol use is lacking [21,23,27]. Our research studies seek to test the effectiveness of alcohol apps for mobile phones in reducing problematic alcohol use in university students, as well what components an efficient app actually needs to include in order to be effective.

The present research project, as well as our prior research studies [26,42], is a collaboration with the student unions at the major universities in Sweden. Our research group has over 20 years experience cooperating with student unions in alcohol research, and our overall experience is that student unions are an effective partner offering both contact information to their members, credibility, and the potential for implementing positive research findings.

The two apps our research team developed for this study are based on several years of experiences in developing and testing face-to-face brief interventions, as well as computerized brief interventions using both desktop computers and interactive voice response (IVR).

### Pros and Cons

In the present project, we decided to use Web-based apps for the two applications our research team developed. This decision has both pros and cons, as Web-based apps are cheap, easy to develop and store user data that is immediately available on our own server, lowering the risk of data losses that may occur when using regular apps run on the mobile phone itself. Regarding cons, users may perceive Web-based apps as a bit slow and sometimes absent, as they are dependent on Internet access. These cons are especially important when comparing the two Web-based apps to a regular app developed by the Swedish retailing company concerning for instance user satisfaction.

## Multimedia Appendix 1

Peer review report from funder (in Swedish).

## Abbreviations

AUDIT: Alcohol Use Disorders Identification Test
BAC: blood alcohol concentration
BASICS: Brief Alcohol Screening and Intervention for College Students
BMI: brief motivational intervention
DDQ: Daily Drinking Questionnaire
eBAC: estimated blood alcohol concentration
HED: heavy episodic drinking
IVR: interactive voice response
PBS: protective behavioral strategies
SBI: screening with brief intervention
WHO: World Health Organization
