# Supplementary material for: Mobile Phone Apps for University Students With Hazardous Alcohol Use: Study Protocol for Two Consecutive Randomized Controlled Trials
Source: JMIR Res Protoc. 2015 Dec 22;4(4):e139. doi: 10.2196/resprot.4894 (PMC4704963; doi:10.2196/resprot.4894)
Supplement: Supplementary file 1 [file resprot_v4i4e139_app1.pdf]

CENTIIALFÖRBUNOET FÖR AJ.l(OHOL· OCH NARKOTIKAUPPLYSNING

12 december 2013

Till docent Ann e H Bcrman

Styrelsen för Systembolagets råd för alkoholforskning (SRA) har vid sitt sammanträde den S december 2013 beslutat bevilja er ett anslag om 200 ooo kronor för projekt 2013-0036 "Mobiltelefoniapplikationer för universitetsstudenter med riskfyllt alkoholbruk ".

Beslutet har fattats efter diskussion i SRAs styrelse och grundar sig på en samlad bedömning av poängsättningen, som gjorts fristående av sty relseledamöterna, samt av innehållet i sakkunnigu tlåtandet (bifogas).

Två original av kontraktet, rekvisitionsblankett samt kontraktsvillkor översändes härmed. Kontraktet skall undertecknas av

- huvudansvarig anslagsmottagare (bevittnas)
- tjänsteman vid förvaltande organ

Det ena kontraktet behålls av huvudansva rig forskare medan det andra insändes till: SRA, CAN, Box 704 12, 107 25 Stockholm. Kopia av undertecknat kontrakt samt kontraktsvillkor lämnas av huvudansva rig forskare till tjänsteman vid förvaltande organ som rekvirerar medlen.

Enligt uppdrag

Britta
Grönlund SRA

Bilagor:
Två originalkontrakt
Rekvisitionsblankett

Kontraktsvillkor
Sakkunnigutlåtande

CAN, Cent .ralförbund et för alkohol· och narkotikaupply sning, www.can.se, info@can. se
adress: Box 70412, I07 25 Stockholm, besöksadress: Klara Norra Kyrkogata 34, tel: 08-4 12 46 00, fax: 08- 10 46 41
bangiro:5626-3841, plusgiro: 30 59-3, organisationsnummer. 802000-5461, bank SEB

SYSTEMBOLAGETS RÅD FÖR ALKOHOLFORSKNING (SRA)
CAN. Box 70412, 107 25 Stockholm. britta.gronlund@can.se

Vetenskaplig bedömning

2013-0036 Doc Anne H Berman
"Mobiltelefoniapplikationer för universitetsstudenter med riskfyllt alkoholbruk"

Frågeställningens originalitet och relevans med anknytning till alkoholforskning
Att studera högskolestudenter med problematiskt bruk av alkohol och effekten av användandet av mobilappar på alkoholkonsumtion. Studie 1. Leder mobiltelefoniappar till minskad alkoholkonsumtion hos studenter med problematisk alkoholkonsumtion i förhållande till kontroller? Studie 2. Leder tillgång till appen TeleCoach till minskad alkoholkonsumtion hos studenter i förhållande till kontroller hos individer som i studie 1 har en riskfylld konsumtion enligt AUDIT efter 6 veckor. De lottas sedan att få använda appen TeleCoach+ korta interventioner i jämförelse med kontroller. Mobiltelefoniappar i relation till alkoholkonsumtion är ett originellt och modernt sätt att försöka minska en riskfylld alkoholkonsumtion på.

Metodik och sökandens vetenskapliga kompetens i förhållande till projektet
Studenter som har ett riskbruk enligt AUDIT lottas till användning av tre olika appar PartyPlanner, Promille koll (Systembolagets egen app) och kontrollgrupp. Deltagarna uppmanas använda appen i samband med fester. De två föreslagna studierna är väl designade med experimentgrupp och kontrollgrupp. Uppföljning sker efter 1, 6 och 12 veckor. Den sökande har en god tidigare forskningserfarenhet, hennes medsökande li kaså. Godkänd etik föreligger samt en ny planerad etik. Projektets totala kostnader uppgår till ca 400 000 kr 2014 varav 300 000 söks av SRA.

Sammanfattande bedömning av vetenskaplig betydelse
Detta projekt ämnar studera om mobiltelefoniappar kan hjälpa till att minska en riskfylld konsumtion av alkohol hos högskolestuderande. Den sökande redogör för två väldesignade studier med experimentgrupp / kontrollgrupp. Hon redogör även för statistisk power analys av studierna. Det finns preliminära resultat som påvisar att apparna Promillekoll och PartyPlanner fungerar till viss nivå. Dessa studier skulle kunna bidra med att man som ung tidigt kan få hjälp med sina alkoholvanor.

Genomförbarhet ur metodologisk och praktisk synpunkt
Ansökan är mycket enkel att förstå. Forskare utanför fältet kan lätt läsa ansökan och förstå den. Det framgår inte att det är ett hypotesdrivet projekt. Däremot anser jag att studierna är vetenskapligt drivna och väl förankrade i metod och genomförbarhet.

Förslag till beviljande eller avslag: Beviljande.
